# The impact of hindlimb disuse on sepsis‐induced myopathy in mice

**DOI:** 10.14814/phy2.14979

**Published:** 2021-07-26

**Authors:** Orlando Laitano, Jose Pindado, Isela Valera, Ray A. Spradlin, Kevin O. Murray, Katelyn R. Villani, Jamal M. Alzahrani, Terence E. Ryan, Philip A. Efron, Leonardo F. Ferreira, Elisabeth R. Barton, Thomas L. Clanton

**Affiliations:** ^1^ Department of Nutrition and Integrative Physiology College of Health and Human Sciences Florida State University Tallahassee FL USA; ^2^ Department of Applied Physiology and Kinesiology College of Health and Human Performance University of Florida Gainesville FL USA; ^3^ Department of Surgery College of Medicine University of Florida Gainesville FL USA

**Keywords:** atrophy, infection, inflammation, muscle, septic shock, weakness

## Abstract

Sepsis induces a myopathy characterized by loss of muscle mass and weakness. Septic patients undergo prolonged periods of limb muscle disuse due to bed rest. The contribution of limb muscle disuse to the myopathy phenotype remains poorly described. To characterize sepsis‐induced myopathy with hindlimb disuse, we combined the classic sepsis model via cecal ligation and puncture (CLP) with the disuse model of hindlimb suspension (HLS) in mice. Male C57bl/6j mice underwent CLP or SHAM surgeries. Four days after surgeries, mice underwent HLS or normal ambulation (NA) for 7 days. Soleus (SOL) and extensor digitorum longus (EDL) were dissected for in vitro muscle mechanics, morphological, and histological assessments. In SOL muscles, both CLP+NA and SHAM+HLS conditions elicited ~20% reduction in specific force (*p *< 0.05). When combined, CLP+HLS elicited ~35% decrease in specific force (*p *< 0.05). Loss of maximal specific force (~8%) was evident in EDL muscles only in CLP+HLS mice (*p *< 0.05). CLP+HLS reduced muscle fiber cross‐sectional area (CSA) and mass in SOL (*p *< 0.05). In EDL muscles, CLP+HLS decreased absolute mass to a smaller extent (*p *< 0.05) with no changes in CSA. Immunohistochemistry revealed substantial myeloid cell infiltration (CD68+) in SOL, but not in EDL muscles, of CLP+HLS mice (*p *< 0.05). Combining CLP with HLS is a feasible model to study sepsis‐induced myopathy in mice. Hindlimb disuse combined with sepsis induced muscle dysfunction and immune cell infiltration in a muscle dependent manner. These findings highlight the importance of rehabilitative interventions in septic hosts to prevent muscle disuse and help attenuate the myopathy.

## Graphical abstract

This study shows the impact of skeletal muscle disuse on sepsis‐induced myopathy in mice. Developed with Biorender software.

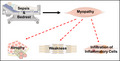

## 1 INTRODUCTION

Sepsis, defined as a life‐threatening organ dysfunction caused by a dysregulated host response to infection, remains a major medical problem worldwide resulting in medical costs exceeding $17 billion per year (Prescott & Angus, 2018). There has been a decrease in the rate of in‐hospital mortality due to sepsis, which is attributed to early recognition and aggressive intensive care unit (ICU) management (Prescott & Angus, 2018; Stevenson et al., 2014). Nevertheless, hospital discharge may not be the end of concerns for sepsis survivors as ~75% of these patients develop persistent sepsis‐induced myopathy (Brakenridge et al., 2018; Callahan & Supinski, 2009; Santos et al., 2016; Steiner & Lang, 2015). The myopathy is characterized by severe and persistent atrophy (e.g., reductions in cross‐sectional area) and weakness (e.g., reductions in force production capacity) in limb and respiratory muscles (Iwashyna et al., 2010; Llano‐Diez et al., 2012; Puthucheary et al., 2013). These abnormalities are strongly associated with mobility impairments affecting the patient's physical activity levels and ability to live independently, resulting in increased re‐hospitalization rates and death within 5 years from the initial insult (Cuthbertson et al., 2013). Although the myopathy has been described over a century ago (Friedrich et al., 2015; Osler, 1912), the impact of other comorbidities present in sepsis, such as limb muscle disuse, remains unknown.

Sepsis survivors often experience prolonged periods of bed rest in the ICU, which may exacerbate the myopathy and lead to greater morbidity and lower quality of life. Currently, there are no effective treatments for this debilitating myopathy. Importantly, current preclinical models available to study the mechanisms of this manifestation do not recapitulate the combination of systemic inflammation and lack of physical activity (e.g., bed rest). Previous attempts to develop rodent preclinical models that mimic ICU conditions to study muscle wasting in critically ill hosts (Larsson, 2007) consisted of long‐term postsynaptic block of neuromuscular transmission to mimic acute quadriplegic myopathy, which is relevant for pharmacologically paralyzed or deeply sedated mechanically ventilated ICU patients (Kalamgi & Larsson, 2016). While this is a relevant model for ICU acquired muscle weakness (also known as critical illness myopathy), it does not apply specifically to septic hosts who undergo immune hyperresponsiveness at the same time as bed rest or the muscle unloading event. To overcome these restrictions, we tested the feasibility of combining a classic model of sepsis (e.g., cecal ligation and puncture [CLP]) with a classic model of disuse (e.g., hindlimb suspension [HLS]) as an alternative model to study sepsis‐induced myopathy in mice. Our goal was to determine the contribution of hindlimb disuse to skeletal muscle abnormalities in septic mice. We assessed muscle mechanics, morphology as well as infiltration of myeloid cells in relevant limb muscles.

## 2 METHODS

### 2.1 Animal care, housing, and study design

We followed the recommendations of the Minimum Quality Threshold in Pre‐Clinical Sepsis Studies (MQTiPSS) for animal care, housing, and study design (Osuchowski et al., 2018; Zingarelli et al., 2019). The study protocol was approved by the Institutional Animal Care and Use Committee at the University of Florida under the numbers 201909372 and 201808822. We studied 26, ~4‐month‐old, C57bl6/j male mice. Animals were purchased from The Jackson Laboratory and housed at the University of Florida Animal Care Facilities under a temperature and relative humidity range of 20–25⁰C and 31%–67%, respectively. Animals were kept under a 12:12‐h light–dark cycle (lights on at 7 a.m. and off at 7 p.m.) and had access to a standard chow (2918–Envigo) and automatic tap water ad libitum. To avoid major variations in the microbiome (Fay et al., 2019), animals were housed in groups of at most five per cage for at least 2 weeks prior to the experiment. Reported information about the procedures conformed to the Animal Research Reporting of in vivo Experiments–ARRIVE guidelines (Kilkenny et al., 2010). Animals were randomly assigned into the following experimental groups: Sham surgery with normal ambulation (*n *= 6; SHAM+NA), sham surgery with hindlimb suspension (*n *= 6; SHAM+HLS), cecal ligation and puncture surgery with normal ambulation (*n* = 6; CLP+NA), and cecal ligation and puncture surgery with hindlimb suspension (*n* = 8; CLP+HLS).

### 2.2 Cecal ligation and puncture and sham surgeries

**FIGURE 1:**
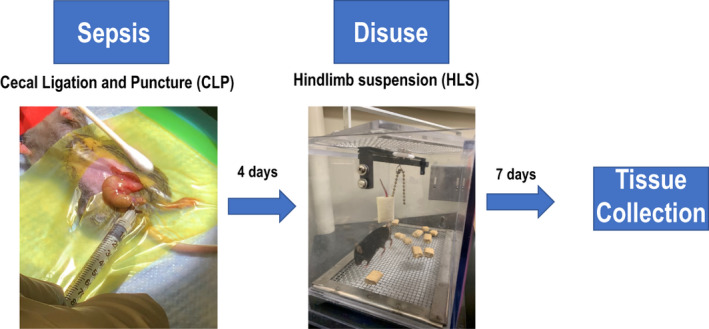
Combination of cecal ligation and puncture (CLP) with hindlimb suspension (HLS) as a pre‐clinical model to study sepsis‐induced skeletal muscle abnormalities. Mice were suspended 4 days after CLP surgeries. HLS lasted for 7 days and muscle tissue was collected for functional and morphological assessment. SOL = soleus, EDL = extensor digitorum longus

We induced intraperitoneal, polymicrobial, and non‐lethal sepsis by performing a modified cecal ligation and puncture (CLP) surgical procedure (Hubbard et al., 2005; Rittirsch et al., 2009). Mice were anesthetized with isoflurane (4% for induction and 1.5% for maintenance in O_2_) for the entire procedure. A small incision was made on the abdominal wall through the skin and muscle layers and the cecum was exposed. The cecum was ligated at the terminal third using 6‐0 monofilament absorbable PGA suture (Demesorb; Demetec). Upon ligation, the cecum was punctured through and through with a 28‐gauge needle (Figure 1). The cecum was immediately returned to its place into the abdominal cavity. For sham surgeries, the cecum was exposed without being ligated or punctured. The abdominal incision was closed, and mice were returned to their cage. Mice were singly caged from this time onwards. To prevent hypothermia, a heating pad set at 37⁰C was placed under the half‐bottom of the cage. Mice were injected with buprenorphine (0.05 mg/kg) and 0.5 cc of saline every 12 h for 2 days and every 24 h for days 3 and 4. To avoid contact with urine scalding and facilitate wound healing from surgeries, mice began the HLS protocol 4 days after surgeries or simply remained with normal ambulation.

### 2.3 Hindlimb suspension

We induced muscle disuse in the hindlimbs of Sham and CLP mice by using a tail suspension model described previously (Park et al., 2013). Four days after sham or CLP surgeries, animals were suspended under light isoflurane anesthesia. Importantly, CLP mice were still septic while undergoing tail suspension. Their tails were attached to a short metal chain via foam tape (Skin Trac; Zimmer). The suspension cages had a crossbar along the center of the cage on which a second small bar could move to allow greater movement ability for the animals. To ensure suspension of the hindlimbs, the metal chain was attached to the small bar and the length was adjusted. Animals were able to move via their forelimbs by utilizing the metal grid on the cage floor and were able to maintain normal activity (Figure 1). Food was made available by adding pellets of chow on the cage floor. We adjusted the height of the suspended limbs to avoid the contact of the paws with the chow pellets. Animals were monitored and hand cleaned at least twice daily during the suspension period. Terminal experiments took place after 7 days of suspension (Figure 1). Sham and CLP time‐matched normal ambulation (NA) mice were included for comparison and were euthanized for analysis as controls. An initial concern was whether septic mice would survive the 7‐day suspension period. All mice survived the experimental period.

### 2.4 Muscle force generation and calculations

At day 7 of suspension, mice were anesthetized with a combination of xylazine (80 mg/kg) and ketamine (10 mg/kg) for the dissection of soleus (SOL) and extensor digitorum longus (EDL) muscles. These muscles were chosen because they are known to express a more pronounced oxidative (e.g., SOL) and glycolytic (e.g, EDL) phenotypes (Augusto et al., 2004), and are in the area subjected to suspension. Muscles were placed in a Sylgard‐based Petri dish, and held at approximately resting length by minuten pins, and bathed in Ringer's solution ([in mM] 120 NaCl, 4.7 KCl, 2.5 CaCl_2_, 71.2 KH_2_PO_4_, 1.2 MgSO_4_, 25 4‐(2‐hydroxyethyl)‐1‐piperazineethanesulfonic acid, and 5.5 glucose) gas equilibrated with 95% O_2_ and 5% CO_2_. Loops on the proximal and distal muscle tendons were tied with nonabsorbable braided silk sutures (Fine Science Tools) and the muscle was transferred into a tissue bath apparatus (Model 800A, Dual Mode Muscle Lever 300C; Aurora Scientific) filled with gas equilibrated Ringer's solution at 22℃. The distal tendon was attached to a rigid post, and the proximal tendon was attached to a force transducer at resting length. The optimal muscle length (Lo) was established by adjusting the muscle length in isometric twitch conditions, until maximum force was obtained. Stimulus frequencies of 5, 15, 30, 50, 80, 120, 150, and 200 Hz (0.25‐ms pulse and 0.5‐s train durations) were studied. For calculation of specific force (force normalized by cross sectional area (CSA), N/cm^2^), CSA was estimated based on muscle mass and length, using the maximum force value attained during the force frequency stimulations. Peak twitch force (N/cm^2^) and half relaxation time (s), which is the time for decay of tension from the peak of the isometric twitch to one half of the peak tension at optimal length, were also determined. After the mechanical procedures, the muscles were blotted, weighed, and rapidly frozen using Tissue‐Tek medium (OCT) submersed in liquid nitrogen‐cooled isopentane and stored at −80℃ for histological analyses. To avoid human bias, researchers performing the muscle mechanics measurements were blinded to the experimental condition.

### 2.5 Muscle cross‐sectional area and myeloid cell infiltration

OCT‐mounted muscles from all four groups were cut in 7‐μm sections using a cryostat (Leica CM1950) and stained with antibodies against dystrophin (MANDYS1(3B7) Development Studies Hybridoma Bank), for cross‐sectional area quantification, and against CD68 (BioLegend, monoclonal mouse IgG2 [anti‐CD68]), for myeloid cell count. All muscle samples were sectioned, immunolabelled, and imaged at the same time and using the same conditions and microscope settings. In brief, muscle sections were fixed in 4% paraformaldehyde in PBS, blocked in 10% goat serum in PBS for 1 h at room temperature. Fixed and blocked sections were then incubated with primary antibodies (e.g., dystrophin and CD68) at a 10 µg/ml dilution in 3% BSA in PBS tween (0.05%) overnight at 4⁰C. Tissue sections were subsequently washed and incubated with fluorophore‐conjugated secondary antibodies at a 1:200 dilution in 3% BSA in PBS for 1 h in the dark at room temperature. Images were acquired using a Leica DMI8 microscope with a 10× objective and a Leica DFC7000T camera. To measure fiber CSA, we analyzed ~1000 fibers per animal and calculated the median area. We traced the membrane boundaries established by dystrophin in all visible fibers of each muscle section using National Institutes of Health ImageJ software with morphoLibJ plugin with manual adjustments. For CD68+ cell count, we used stitched images to quantify the infiltration in the entire section of SOL and EDL muscles. Positively labeled cells were counted using Image J software.

### 2.6 Statistical analysis

All data are presented as mean ± SE. Statistical analyses were performed using GraphPad Prism v. 9.0 for Windows (GraphPad Software) and SAS JMP V.16. Data distributions were tested for normality using the Shapiro–Wilk test. We used two‐way ANOVA with multiple comparisons and, where appropriate, Tukey's test for post hoc comparisons. For analysis of the force frequency curves we used best fit using sigmoidal Hill equation. Differences were considered statistically significant when *p* < 0.05.

## 3 RESULTS

### 3.1 Disuse impacts body mass and exacerbates sepsis‐induced atrophy

**FIGURE 2:**
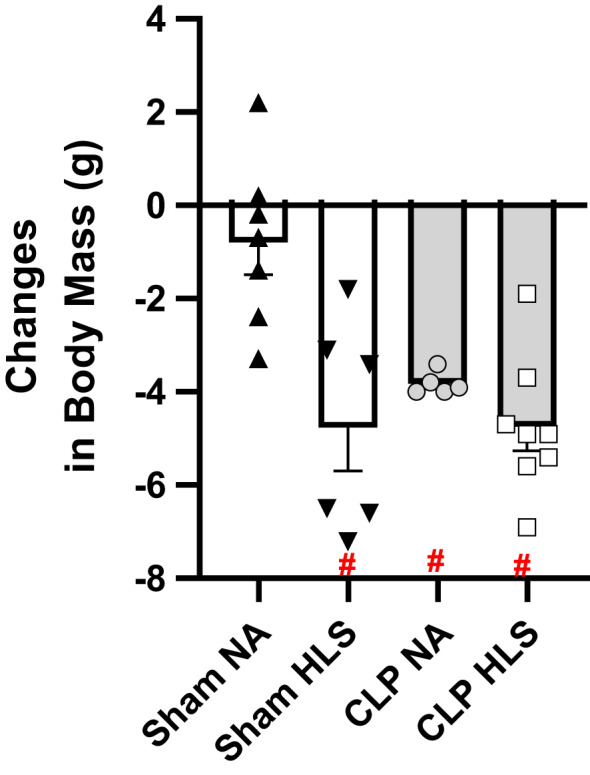
Changes in body mass induced by each experimental intervention. ^#^ *p *< 0.05 in comparison to Sham+NA group. Data are mean and standard error (SE). Two‐way ANOVA with multiple comparisons

To determine the impact of sepsis and disuse on total body mass, we analyzed animals’ body mass at the beginning and at the end of the experimental period. Initial body mass, recorded before surgeries, was similar among the groups (SHAM+NA = 31.1 ± 1.3 g; SHAM+HLS = 31.4 ± 1.4 g; CLP+NA = 29.6 ± 1.4 g; CLP+HLS = 29.5 ± 1.2 g). We did not record animals’ body mass throughout the HLS period to avoid having the suspended animals touch the ground surface. As shown in Figure 2, all experimental conditions (SHAM+HLS, CLP+NA, and CLP+HLS) resulted in similar reductions in body mass, in comparison to SHAM+NA control condition, ranging on average from 4 to 5 g (*p* < 0.05). These observations indicate that both disuse and sepsis result in marked total body mass loss, though the combination of sepsis with disuse did not result in further reductions in total body mass in this model.

**FIGURE 3:**
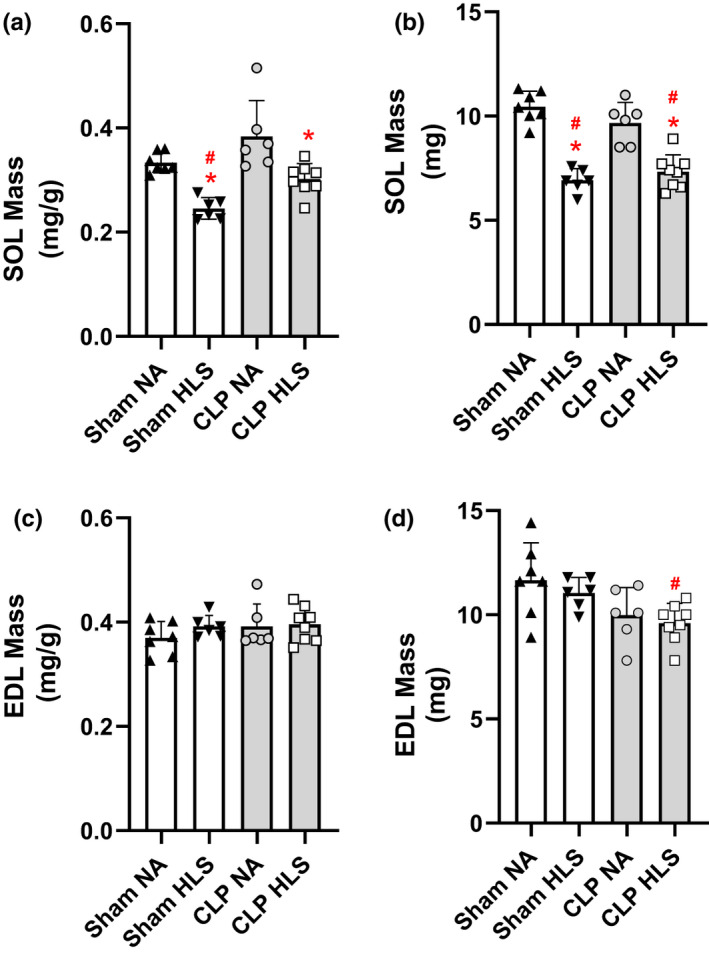
Muscle mass for SOL (panels a & b) and EDL (panels c & d) normalized by body mass (mg/g) and expressed as absolute (mg). Data are mean and standard error (SE). Two‐way ANOVA with multiple comparisons. ^#^ different from Sham+NA; * different from CLP+NA

**FIGURE 4:**
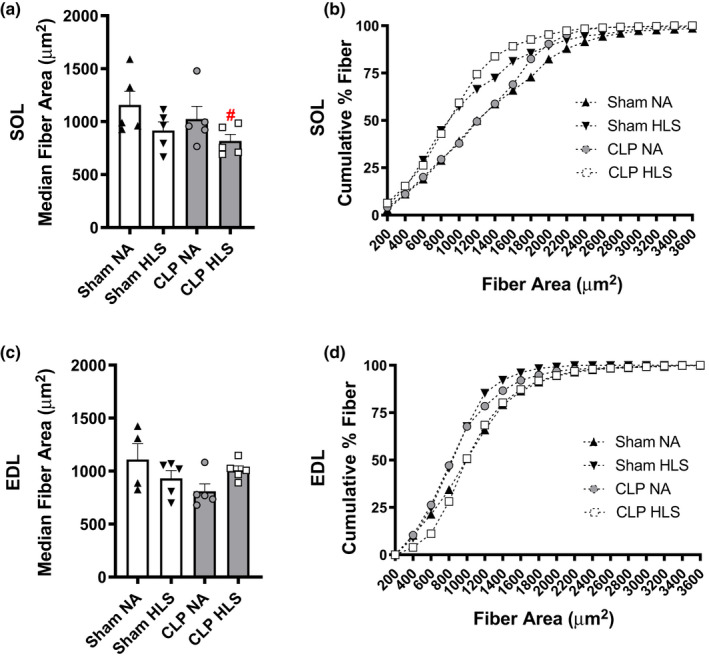
Median fiber areas show that CLP+HLS resulted in atrophy in SOL (panel a), but not in EDL muscles (panel c). Data are median and standard error (SE). Two‐way ANOVA with multiple comparisons. Cumulative percentage fiber size distributions highlight the leftward shift of the fiber area distribution, indicating that a greater proportion of fibers were smaller (panels b and d). ^#^ different from Sham+NA

To address whether sepsis and disuse resulted in muscle atrophy, we measured skeletal muscle mass and analyzed it in relative (normalized by body mass) and absolute terms. As shown in Figure 3, a decrease in both relative (mg/g) and absolute (mg) SOL mass was observed in SHAM+HLS and CLP+HLS (*p* < 0.05). EDL muscles only presented loss of mass, in absolute terms, when sepsis and disuse (CLP+HLS) were combined (*p* < 0.05 vs. SHAM+NA). As shown in Figure 4, the loss of muscle mass was the result of reductions in median muscle fiber area (Figure 4a), as illustrated by the leftward shift of the cumulative distributions of fiber area of SOL (Figure 4b). For EDL muscles, no changes were observed in CSA (Figure 4c, d).

### 3.2 Disuse exacerbates sepsis‐induced weakness

**FIGURE 5:**
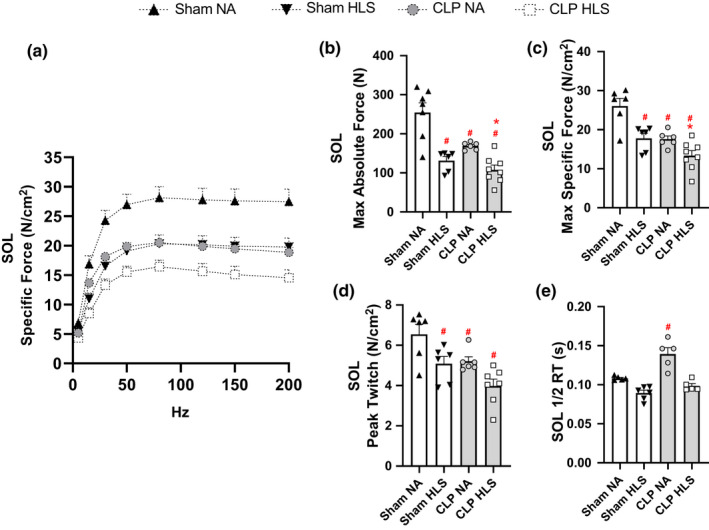
Soleus (SOL) (panel a) force‐frequency curves. Lines represent best fit using sigmoidal Hill equation. SOL maximal absolute (panel b) and specific forces (panel c); peak twitch (panel d) and half‐relaxation time (Panel e). SOL maximal specific force was markedly reduced in SHAM+HLS and CLP+NA mice and further reduced in CLP+HLS mice. Peak twitch was reduced in SHAM+HLS, CLP+NA, and CLP+HLS mice. ^#^ different from Sham+NA; * different from CLP+NA

To establish the impact of sepsis and disuse on muscle force generation capacity we determined specific force over a range of stimulation frequencies for SOL and EDL muscles in each experimental condition. SOL maximal specific force was markedly reduced by ~20% in both SHAM+HLS and CLP+NA groups. Combined CLP+HLS resulted in ~35% decrease in maximal specific force (Figure 5a–c). Peak twitch was decreased in SHAM+HLS, CLP+NA, and CLP+HLS mice (*p* < 0.05) (Figure 5d). Half relaxation time was significantly longer in CLP+NA (*p* < 0.05) (Figure 5e).

**FIGURE 6:**
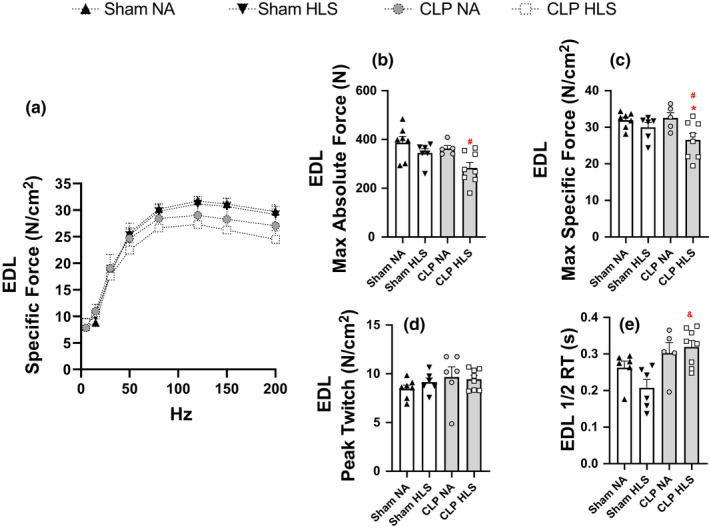
Extensor digitorum longus (EDL) (panel a) force‐frequency curves. Lines represent best fit using sigmoidal Hill equation. EDL maximal absolute (panel b) and specific (panel c) forces; peak twitch (panel d) and half‐relaxation time (panel e). EDL force was only reduced by the combination of CLP+HLS. Data are mean and standard error (SE). Two‐way ANOVA with multiple comparisons. ^#^ different from Sham+NA; * different from CLP+NA; ^&^ different from Sham+HLS

For EDL muscle, we only observed a decrease of 8.5% in maximal force when CLP+HLS were combined (Figure 6a–c). No changes were observed in peak twitch in EDL muscles (Figure 6d). Half relaxation time was longer in CLP+HLS than SHAM+HLS mice (*p* < 0.05) (Figure 6e). Overall, these observations indicate that disuse aggravates sepsis‐induced weakness in SOL, and at a smaller magnitude in EDL muscles.

### 3.3 Disuse promotes myeloid cell infiltration in SOL muscles of septic mice

**FIGURE 7:**
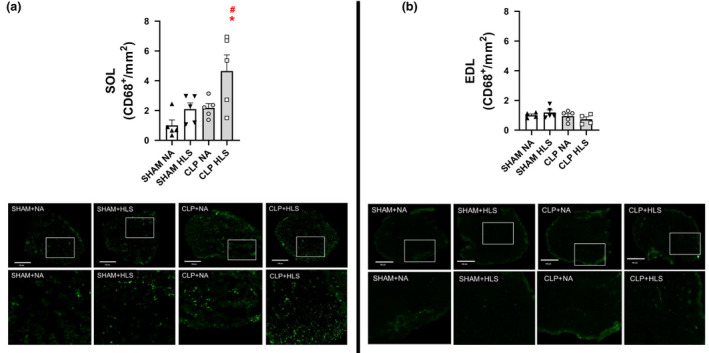
Increased infiltration of CD68+ cells was observed in SOL muscle (panel a) in the CLP+HLS group. No changes were observed in EDL muscles (panel b). Data are mean and standard error (SE). Two‐way ANOVA with multiple comparisons. Representative sections of SOL and EDL depicting the greater accumulation of macrophages (in green) in CLP+HLS condition for SOL muscles. White squares at the top panels represent areas of magnification in the bottom panels. ^#^ different from Sham+NA; * different from CLP+NA

To establish whether sepsis and disuse would alter the population of macrophages (CD68+ cells) we performed immunohistochemistry in SOL and EDL sections (Figure 7). We observed a marked accumulation of CD68+ cells in SOL (Figure 7a) muscles in CLP+HLS conditions (*p* < 0.05). We did not observe any changes in EDL muscles (Figure 7b). These observations indicate that disuse, when combined to sepsis, promotes the accumulation of inflammatory cells in SOL muscles as illustrated in representative cross‐sectional images in Figure 7.

## 4 DISCUSSION

The main finding of this study is that the combination of sepsis, via CLP, and disuse, via HLS, is a feasible model to study sepsis‐induced myopathy with disuse in mice. To our knowledge this is the first study to report the impact of disuse via HLS on skeletal muscle abnormalities in septic mice. Our data support the notion that hindlimb muscle disuse exacerbates skeletal muscle weakness, atrophy, and infiltration of myeloid cells in muscles. This is important in the context of sepsis because muscle disuse is a common comorbidity faced by septic patients undergoing hospitalization. Thus far, the majority of preclinical studies addressing sepsis‐induced myopathy did not include disuse via unloading as a feature (Owen et al., 2019; Supinski et al., 2020). The design of intervention strategies to attenuate muscle wasting and weakness in septic hosts requires models that mirror, more closely, the clinical features of the disease. Our observations indicate that the combination of CLP and HLS translate the clinical events experienced by a hospitalized septic patient who survive the initial insult and experience physical convalescence due to muscle disuse (i.e., in the absence of pharmacological paralysis and sedation).

The combination of CLP with HLS, as a preclinical model to study sepsis‐induced myopathy with disuse, has some unique advantages that are worth highlighting. First, it results in two comorbidities often seen in septic patients (e.g., immune hyperresponsiveness and muscle disuse). Second, the animals are still septic during the 7‐day period of disuse, considering that CLP induces sepsis in mice for up to 28 days after surgeries (Laudanski et al., 2017). Third, the abnormalities seen in skeletal muscles closely reflect previous observations in muscles from human septic patients. For instance, we observed a marked muscle wasting and weakness induced by the combination of CLP and HLS. These findings are consistent with data from Puthucheary et al. (2013) in human septic patients with multi‐organ dysfunction reporting progressive muscle wasting and accumulation of CD68+ cells over the course of 7 days. Although these authors did not report the time spent in bedrest, the multiorgan dysfunction assessment suggests that those patients might have undergone physical inactivity for prolonged periods of bedrest. Therefore, our alternative model combining CLP with HLS may offer an additional tool to perform hypothesis‐driven research to resolve the mechanisms underlying the muscle dysfunction in septic, physically inactive patients. These features represent a novel and unique alternative to study regulation of myofibrillar protein synthesis/degradation, regulation of muscle contraction, muscle structure, muscle size, and effects of different interventions (e.g., genetically, or pharmacologically) on muscles over an extended period of time in the context of sepsis.

The skeletal muscle system is the “forgotten” organ‐system affected by sepsis despite observations that muscle abnormalities occur in human and murine sepsis (Owen et al., 2019; Puthucheary et al., 2013). Besides locomotive reasons, which include the ability to live a functional life, there are several ways in which skeletal muscles participate in immune function in response to sepsis and other stress‐induced illnesses. For example, skeletal muscles serve as a cytokine response organ during muscle contraction and exposure to pathogen‐ or damage‐associated molecular patterns (PAMPS and DAMPS) or in response to cellular stress (Laitano, Robinson, Garcia, et al., 2021; Laitano, Robinson, Murray, et al., 2021; Lang et al., 2003; Welc et al., 2016). Skeletal muscles can also serve as a source of acute phase proteins (APPs) (Iwaniec et al., 2021; Langhans et al., 2014) and amino acid substrates to support highly metabolic tissues such as liver, kidney, and bone marrow (Karinch et al., 2001), and as part of a complex network of resident immunosensitive cells that reside together within muscle bundles (Côt́e et al., 2008; Howard et al., 2020; McLennan, 1993). Thus, it is likely that muscle wasting and weakness in sepsis survivors result not only in poor functional capacity but also in compromised immune response to cope with subsequent insults. Rehabilitative strategies to prevent disuse in septic hosts may preserve muscle function (Mankowski et al., 2021), although this may be a challenging intervention in severe septic conditions.

Most of our current knowledge about the potential mechanisms underlying sepsis‐induced myopathy comes from studies focusing on respiratory muscles in models of endotoxic shock and peritonitis (Maes et al., 2014; Supinski et al., 1985, 2020). Maes et al. (2014) induced endotoxic shock by injecting lipopolysaccharide (LPS) in rats undergoing 12 h of mechanical ventilation to induce diaphragm unloading. They reported exacerbation of diaphragm dysfunction induced by the combination of LPS and diaphragm inactivity, which was likely induced by increased cytokine production, autophagy, and oxidative stress. No exacerbation of atrophy was observed, likely due to the relatively short period of intervention (24 h). It is challenging to draw parallel comparisons between Maes et al. and our study for several reasons. First, the infection was induced by LPS injection in their study. LPS is the most abundant component within the cell wall of gram‐negative bacteria, but LPS injection results in endotoxic shock and not sepsis. In our study we induced sepsis via CLP, which is a polymicrobial form of peritonitis. Second, Maes et al. studied the diaphragm muscle 24h after the LPS injection, whereas we studied limb muscles 11 days post CLP surgeries. Third, Maes et al. induced diaphragm unloading via mechanical ventilation for 12 h, whereas in our study we induced hindlimb disuse via tail suspension for 7 days. Their study confirms earlier observations that overproduction of reactive oxygen species (ROS) may be a mechanism involved in muscle weakness when endotoxemia and inactivity are involved. In their review, Callahan and Supinski (2009) proposed that mechanisms governing sepsis‐induced myopathy include circulatory cytokines, enhanced ROS generation in muscle, increased muscle proteolysis and decreased muscle synthesis. The additional loss of external strain related to the absence of weight bearing stimuli further enhanced the loss of force induced by sepsis in muscles in our model. In addition, we observed that SOL muscles display atrophy, loss of force, and accumulation of macrophages. It is reasonable to expect that accumulation of macrophages and other inflammatory cell populations would result in higher production of ROS. High ROS production is associated with halted protein synthesis and accelerated protein degradation due to enhanced proteasome proteolytic degradation and autophagy pathways (Callahan & Supinski, 2009; Powers et al., 2012; Preau et al., 2019; Wollersheim et al., 2014). Whether and when these pathways are upregulated over the course of the myopathy, in our double‐hit model, warrants further investigation.

Oxidative muscles seem to be highly susceptible to abnormalities induced by sepsis. Preau et al. have identified a necrotizing phenotype in SOL muscle of rats exposed to a model of peritonitis in contrast to the glycolytic gastrocnemius muscle, which did not display the necrotic phenotype (Preau et al., 2019). Owen et al. (2019) demonstrated that sepsis, induced via cecal slurry injection, caused EDL weakness, but not atrophy. In contrast, Rossignol et al. (2008) demonstrated that CLP performed in rats induced loss of EDL CSA and a 30% decrease in maximal twitch force 10 days after surgery. Importantly, none of the abovementioned studies incorporated muscle disuse in the septic model. At this point it is difficult to determine whether the different responses between SOL and EDL muscles, in our model combining sepsis with disuse, could be attributed to fiber‐type specificity (e.g., glycolytic vs. oxidative) or simply because of the biomechanistic role played by these muscles. For instance, the EDL muscle is a feather‐like muscle of the anterior (extensor) compartment of leg, the origins of which are at the proximal half of medial surface of fibula, lateral tibial condyle, and interosseus membrane (Chleboun et al., 1997). The muscle inserts into the distal and middle phalanges of digits 2–5. Since the EDL crosses the dorsal aspect of the ankle joint, its common function is dorsiflexion of the foot/paw. During HLS, the EDL tends to be more active as the animals concentrically contract the EDL muscles as an attempt to reach the ground. On the other hand, mechanical loading of muscle tissue is a major component of SOL muscle activity (e.g., plantar flexion), which is directly compromised by the HLS. Others have supported the notion that EDL muscles are less affected by HLS than SOL muscles (Haida et al., 1989).

Another potential explanation for the observation of greater abnormalities in the SOL muscle could be the abundance of toll‐like receptors (TLRs) that is known to be greater in SOL muscles (Pillon & Krook, 2017). Muscles sense and respond to circulating pathogens and signals arriving from damaged cells via receptors, particularly TLRs on the sarcolemma (Kawasaki & Kawai, 2014). All membrane bound TLRs, such as TLR4, as well as interleukin (IL)‐1β signal through an adapter protein, myeloid differentiation primary response 88 (MyD88), which eventually activates downstream nuclear factor κB (NF‐ƘB) signaling networks (Kawasaki & Kawai, 2014). NFƘB is a central player in intracellular inflammatory responses and is a critical link between inflammation and most forms of muscle atrophy and myopathy. Therefore, it is possible that the greater abundance of TLRs in SOL muscle could partially explain why this muscle is affected by our model at a greater extent. Our observations of altered half‐relaxation time may indicate that calcium (Ca^2+^) dysregulation may be involved in the myopathy and this warrants further investigation.

Although our present study is the first to show the impact of disuse, via HLS, in septic mice and to propose an alternative double‐hit model combining CLP with HLS to study sepsis‐induced myopathy, our study is not without limitations. Sepsis incidence is similar between males and females. Data from prospective clinical studies indicate that 54% of septic patients admitted to ICU over the course of a year were males and 46% were female (Nasir et al., 2015). We only studied male mice in the present study and it is well known that there are different responses between male and female mice regarding the skeletal muscle response in the context of sepsis (Laitano, Robinson, Garcia, et al., 2021; Laitano, Robinson, Murray, et al., 2021). In addition, we did not monitor food intake in the present study. Food intake has a major effect on body composition in mice (Yan et al., 2011) and could influence muscle size, contractility, and function in general. While we recognize that determining food intake with precision in mice is challenging (Starr & Saito, 2012), in particular when pelleted or ground chow is used, we appreciate that future experiments should account for the potential impact of changes in food intake on the myopathy. Sepsis is considered the disease of the aged as 60% of septic patients are older than 65 years (Kumar et al., 2011; Turnbull et al., 2009), yet in the current study we studied young adult mice. One concern with double‐hit models using aged mice (>20 months) is whether they would tolerate the stress of the model. Indeed, we are currently exploring this model in cohorts of older mice and preliminary data suggest great tolerance to the model with low mortality rates in aged mice. Though studies to address these gaps in knowledge regarding our model are underway, the results herein described are limited to young adult, male mice.

In conclusion, our data show that combining CLP with HLS is a viable model to study sepsis‐induced myopathy in mice. In addition, our data confirm that hindlimb disuse aggravates sepsis‐induced muscle abnormalities in male mice with a greater impact in muscles with predominantly oxidative fibers. Moreover, inflammatory cell infiltration may mediate the additive impact of disuse to the sepsis‐induced myopathy in oxidative muscles. These findings highlight the importance of rehabilitative interventions in septic hosts to prevent muscle disuse and help attenuate the myopathy.

## CONFLICTS OF INTEREST

Authors declare that they have no conflict of interest to disclose.

## AUTHOR CONTRIBUTIONS

OL: Conception of the idea, designed the research, performed the experiments, analyzed and interpreted the data, leadership of experiments, and wrote the first draft of the paper. JP: Analyzed the data and reviewed the paper. IV: Analyzed the data and reviewed the paper. RAS: Performed the experiments and reviewed the paper. KOM: Performed the experiments, analyzed the data, and reviewed the paper. KRV: Analyzed the data and reviewed the paper, JMA: Performed the experiments and reviewed the paper. TER: Conception of the idea and reviewed the paper. PAE: Conception of the idea and reviewed the paper. LFF: Conception of the idea and reviewed the paper. ERB: Conception of the idea, designed the research, interpreted the data, and reviewed the paper. TLC: Conception of the idea, designed the research, and reviewed the paper.
